# Critical cell wall hole size for lysis in Gram-positive bacteria

**DOI:** 10.1098/rsif.2012.0892

**Published:** 2013-03-06

**Authors:** Gabriel J. Mitchell, Kurt Wiesenfeld, Daniel C. Nelson, Joshua S. Weitz

**Affiliations:** 1 School of Biology, Georgia Institute of Technology, Atlanta, GA, USA; 2 School of Physics, Georgia Institute of Technology, Atlanta, GA, USA; 3 Department of Veterinary Medicine, Institute for Bioscience and Biotechnology Research, University of Maryland, Rockville, MD, USA

**Keywords:** enzybiotic, biophysics, membrane dynamics, microbiology

## Abstract

Gram-positive bacteria can transport molecules necessary for their survival through holes in their cell wall. The holes in cell walls need to be large enough to let critical nutrients pass through. However, the cell wall must also function to prevent the bacteria's membrane from protruding through a large hole into the environment and lysing the cell. As such, we hypothesize that there exists a range of cell wall hole sizes that allow for molecule transport but prevent membrane protrusion. Here, we develop and analyse a biophysical theory of the response of a Gram-positive cell's membrane to the formation of a hole in the cell wall. We predict a critical hole size in the range of 15–24 nm beyond which lysis occurs. To test our theory, we measured hole sizes in *Streptococcus pyogenes* cells undergoing enzymatic lysis via transmission electron microscopy. The measured hole sizes are in strong agreement with our theoretical prediction. Together, the theory and experiments provide a means to quantify the mechanisms of death of Gram-positive cells via enzymatically mediated lysis and provides insights into the range of cell wall hole sizes compatible with bacterial homeostasis.

## 1. Introduction

Despite intensive study of the bulk properties and molecular composition of Gram-positive bacterial cell walls [1–4], there is remarkably little first-principles-based theory that considers the effect of defects (e.g. holes) on a cell's viability. Quantifying the effects of defects is of interest from a basic biophysical perspective, but also holds practical relevance in the development of antimicrobial therapeutics. The emergence of antibiotic-resistant bacteria [5–9] has spurred the development of alternative antimicrobials, including metabolites, peptides and enzymes that target cell surfaces [10–12]. One class of antimicrobial enzyme, cell wall hydrolases, cleaves bonds in the cell wall and ultimately induces cell death through bacteriolysis [13–16]. Despite their utility, more detail of the mechanisms by which cells are lysed remains unclear, for example, the identity of target receptors and critical size of defects. Understanding these mechanisms could enable improvements to antimicrobial therapeutics.

Recently, a biophysical theory of defects in cell walls of Gram-negative bacteria was proposed to understand how defects in cell surfaces could lead to lysis [17]. The central theoretical prediction was that sufficiently large holes in the cell walls of Gram-negative bacteria could arise that will lead to protrusion of the membrane and eventually lysis of the cell. However, the structure of Gram-negative bacteria and Gram-positive bacteria differs significantly and it remains unclear how fundamental differences in cell surface properties determine the susceptibility of bacteria to exogeneous lysis. The cell walls of Gram-positive bacteria are composed of a complex network of peptidoglycan along with covalently bound carbohydrates and cell wall associated proteins [2,18,19]. This cell wall extends as far as 50 nm from the cell's membrane and may represent as much as 25 per cent of the dry mass of the cell with peptidoglycan and non-peptidoglycan constituents represented in approximately equal mass fractions [20–22]. This can be contrasted with the cell walls of Gram-negative bacteria that have typical cell wall thicknesses of 5–10 nm with only 10 per cent of that composed of the stress bearing peptidoglycan. Indeed, existing work on modelling the effects of cell wall defects in Gram-negative bacteria assumes a one-dimensional network of peptidoglycan strands [23,24]. Finally, the cell wall constitutes the outer layer of Gram-positive bacteria, whereas the cell wall lies between the inner and outer membrane of Gram-negative bacteria and is thus protected from direct exposure to the environment. These essential differences must be taken into account in developing models of bacterial lysis.

Here, we develop a quasi-static biophysical theory of the membrane profile in response to a hole in the cell wall of a Gram-positive cell. We explicitly account for the finite thickness of the cell wall and the high pressure inside the cytoplasm. After introducing the model, we perform a bifurcation analysis to predict a critical hole size in the range of 15–24 nm beyond which a cell will lyse. The prediction is first compared and shown to be consistent with prior experimental measurements of hole sizes, smaller than our predicted critical hole size, in viable cells. We then test the theory by measuring hole sizes in populations of *S. pyogenes* undergoing lysis after exposure to the most potent phage lytic enzyme identified and characterized to date: PlyC [25]. Measurements of hole sizes range from 22 to 180 nm, serving to validate our prediction with no additional free fitting parameters. We conclude by discussing extensions to the model and future experiments that could facilitate understanding of the fundamental mechanisms of lysis.

### 2. Biophysical model

The starting point of our theory is the Gibbs free energy of the membrane–cytoplasm system at constant pressure and the boundary conditions set by a rigid cell wall which is written as2.1
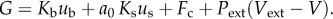


Here *K*_b_ and *u*_b_ are the bending rigidity (units of energy) and specific bending energy (dimensionless) of the membrane, respectively. Likewise *a*_0_, *K*_s_ and *u*_s_ are the initial surface area of the cell membrane, the area stretch modulus (units of energy per unit area) and specific stretching energy (dimensionless) of the membrane, respectively. *F*_c_ is the Helmholtz free energy of the cytoplasm, *P*_ext_ the external pressure and *V*_ext_−*V* is the difference between the volume of the external container and the volume contained inside the membrane. The specific rigidity and bending energy can be written as2.2
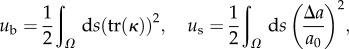
where *Ω* and d*s* respectively denote the membrane surface and infinitesimal surface element, *κ* is the curvature tensor [26,27] and *Δa* is the change in the membrane surface area.

**Figure 1.**
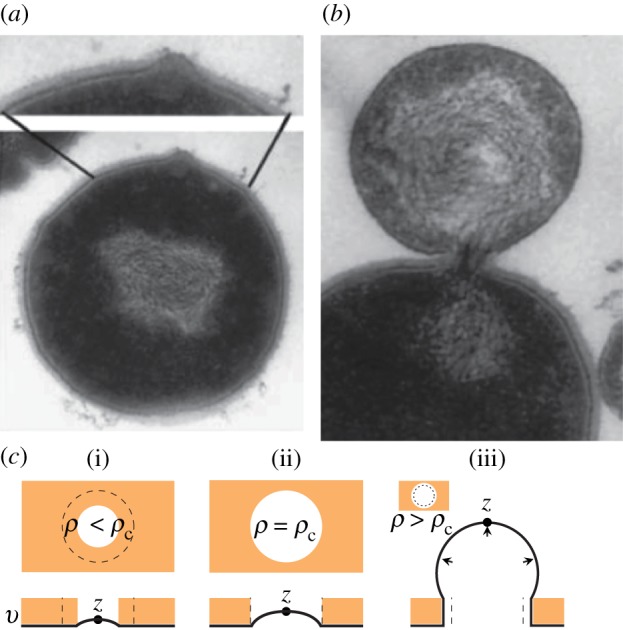
(*a*) An image [29] of the bulging of the membrane of a *Streptococcus* spp. cell after the emergence of a hole in the cell wall owing to the action of lytic enzymes. (*b*) An image of the cell membrane protruding from a cell undergoing lysis. Further stretching of the membrane eventually leads to rupture of the bilayer [30], and the contents of the cytoplasm leak into the environment. (*c*) An illustration of the cavity geometry and equilibrium membrane profiles at subcritical (i) and critical (ii) values of *ρ*. In (iii), *ρ* is supercritical, and the membrane is mechanically unstable, which leads to lysis. (Online version in colour.)

In principle, these energies can be calculated for an arbitrary membrane profile, but exact solutions of the minimum free energy profile require solving a fourth-order nonlinear differential equation obtained from the first moment of variation of the free energy functional, which is, in general, intractable [28]. As such, we focus on analysing the minimum free energy within a restricted geometry observed in prior experiments, consisting of a spherical cap and cylindrical stalk protruding with maximum displacement *z* through a cylindrical cavity of radius *ρ* and height υ (figure 1 and electronic supplementary material, and appendices A–B). The height υ corresponds to the thickness of the cell wall. Given these geometric constraints and assuming a constant 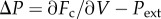, we compute the total Gibbs free energy to arrive at equation (2.3). Equation (2.3) can be used to calculate the generalized force, −*G*_*z*_(*z*,*ρ*).2.3
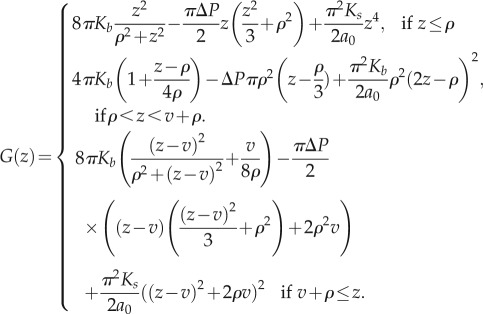

### 3. Model analysis and predictions

**Figure 2.**
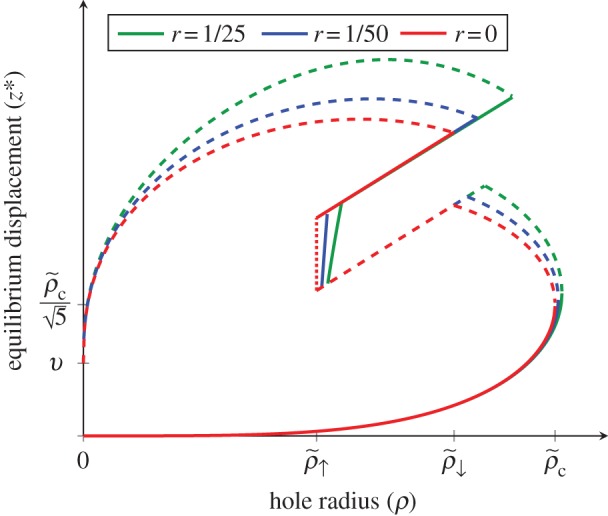
The bifurcation diagram of the stable (solid line) and unstable (dashed lines) fixed points and the marginally stable interval (red dotted line) for finite cell wall thickness υ and various values of 
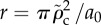
. *K*_b_, *K*_*s*_ and *ΔP* are held constant throughout. (Online version in colour.)

Changes in the configuration of the membrane in response to a hole formed in the cell wall reflect the varying strengths of pressure, bending and stretching forces. The force terms associated with the bending and stretching of the membrane will tend to pull the membrane inwards. The pressure associated force term pushes the membrane outwards. When these forces are balanced, as illustrated in figure 2*a*, the membrane has an equilibrium. The equilibria for a given *ρ* are obtained by solving numerically for *G*_*z*_(z*, *ρ*) = 0, with the corresponding stabilities given by sgn(*G*_*zz*_(z*, *ρ*)) as in figure 2. The number of pairs of stable and unstable fixed points depends on the hole radius *ρ*, and there are several critical radii at which pairs of stable and unstable equilibria are created and destroyed. A relative measure of the potential effect of the stretching term is described by the ratio between the initial critical hole area and the total membrane area 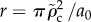. All of the diagrams are qualitatively similar to the *r* = 0 case, which ignores the forces associated with stretching. As such, we discuss this case in detail and comment afterwards on the effect of stretching.

In figure 2, we observe a sequence of three critical radii that we denote as 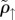, 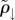 and 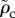 for *r* = 0. Here, the ↑ subscript denotes the creation of a pair of stable and unstable equilibria at the base and top of the hole. Likewise, the ↓ denotes annihilation of the upper unstable equilibria with the stable equilibria at the top of the hole. The critical point 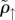 is determined by finding the hole size above which the force owing to pressure in the region 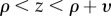 exceeds the force owing to bending in the same. Both forces are constant in this region, and the condition of equal pressure and bending forces (see equation (2.3)) yields the equation 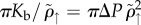. The critical point 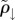 can be determined by finding the minimum hole size at which the force at 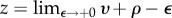 is equal to the force at 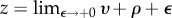. This yields the equation 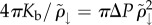. From the above, it follows that3.1
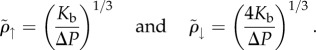
To derive the critical radius 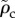, it is useful to define the non-dimensionalized version of the force equation3.2
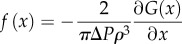
3.3
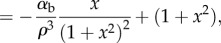
where 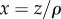 and 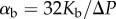. Setting *f*(*x*) = 0, we have3.4
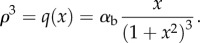


The right-hand side tends to zero in the limit of large, and small *x* and has a single maximum 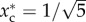. Thus, for sufficiently small *ρ*, there are exactly two real solutions to equation (3.4), and no real solution for 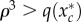, from which it follows that 
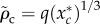
. We obtain3.5
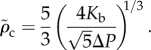


Notably, all of the three critical values are independent of *v*, a point to which we return to later. Moreover, the 1/3 power law dependence of the critical values with respect to the ratio of the bending rigidity, and the pressure difference is consistent with naive expectations from dimensional analysis in the limit that membrane stretching energy and cell wall thickness go to zero. Physically, the point 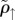 corresponds to the minimum hole size beyond which the specific pressure–volume work exceeds the specific bending energy of a cylindrical membrane. The stable fixed point created at 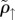 persists until 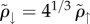, because the force required to push out a spherical bulge is greater than the force required to push out a cylindrical bulge (in the model, it is greater by a factor of 4). The rate of change of the force at the origin is 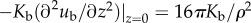 so that the bending force locally is approximately the product of 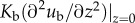 and the displacement. The rate of change of the pressure force at the origin is zero, so we can approximate it as the constant 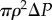. As such we estimate, the stable fixed point near the origin as 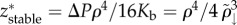. From this, we can calculate the free energy barrier between this equilibria and the unstable equilibria at 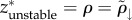 as3.6
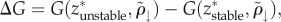
3.7
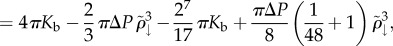
3.8
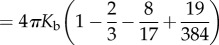
which is about 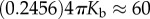
–120 kT. We note that this large barrier is pressure-independent, so that stochastic jumping from the small displacement stable branch onto the large displacement stable branch is suppressed generically.

The earlier-mentioned analysis reveals that 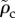 is the physically meaningful critical hole radius beyond which lysis occurs in the absence of stretching associated forces. We note that the factor 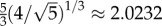 is consistent with Daly's numerical estimate of the same (≈ 2) in the case of Gram-negative cells [17]. Accounting for the contribution of the finite thickness of the membrane bilayer to the hole size 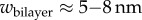, 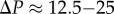 atm [31,32] and 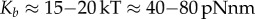 [33], our minimal model estimates a range of observed critical hole diameters 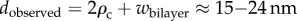. This prediction assumes no effect of stretching.

To investigate the effects of stretching on the final critical value *ρ*_c_ (the absence of the tilde indicates that the expression holds for *r* ≥ 0), we analyse the non-dimensionalized equation for the force3.9
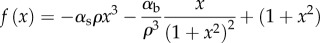
with 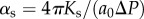. Treating *α*_*s*_ as a small parameter, we propose solutions of the form 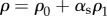. Substituting this into equation (3.9) and letting *f*(*x*) = 0 yields3.10
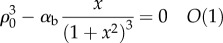
and3.11
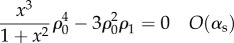
from which we obtain3.12
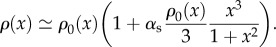
For small *α*_s_, the new maximum will occur at a point 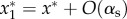. To leading order in *α*_s_, we can evaluate equation (3.12) at x*, which yields3.13
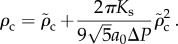
The perturbative correction has a length-scale set by 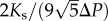, with the magnitude of the correction increasing with the ratio of the hole area and the membrane equilibrium area 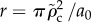. The largest possible contribution from the perturbative term in this regime is less than 1 nm, assuming a spherical bacterium of radius 500 nm and 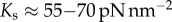 [33], which we take as justification for our disregarding stretching at naturally occurring pressure differences.

## 4. Comparison with experiments

Our prediction for the critical hole diameter can be compared with measurements and theoretical estimates of hole sizes of unlysed cells and hole sizes of lysed cells. For the former, diffusion-based assays [34] indicate that the mean hole diameter in the Gram-positive *Bacillus subtilis* is 2.9–5.5 nm. An alternative method using measurements of the pore widths of conserved secretion machinery leads to estimates of 6.5 nm, a factor of two smaller than the lower end of our estimate [35]. Finally, Meroueh *et al.* [36] chemically synthesized a Gram-positive peptidoglycan strand, solved the structure by NMR and constructed an estimate of naturally occurring pore size of 7 nm from an *in silico* model based on the solved structure. We are not aware of any measurement of a hole diameter in a live Gram-positive bacterial cell larger than our estimate for the critical hole diameter.

**Figure 3.**
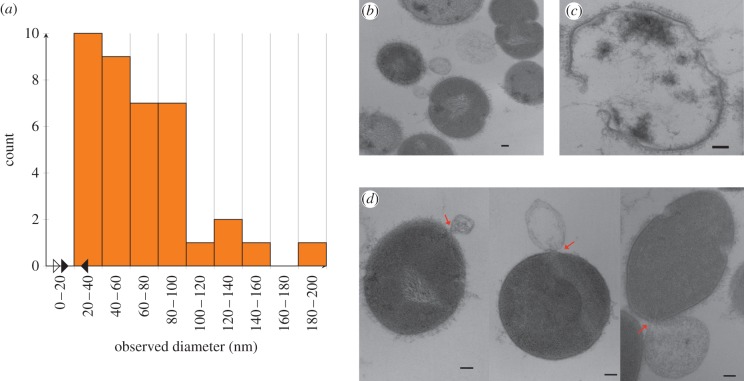
(*a*) The distribution of cell wall hole diameters (bars) after lysis obtained from EM images. The hollow arrow marks the largest estimated cell wall hole diameter for intact cells (7 nm). The two solid arrows indicate the predicted range of critical hole diameters (15–24 nm) that contains the smallest measured diameter of 22 nm. (*b*) An image of a field of cells, demonstrating variability in the timing of bursting events. (*c*) The husk of a cell wall after lysis, showing intact fragments and the gross absence of the cytoplasm. (*d*) Example images showing membrane-bursting events for small (32 nm), medium (47 nm) and large (81 nm) hole diameters. (*b*–*d*) The scale bars are 100 nm in each case. (Online version in colour.)

We further tested predictions of the model by measuring hole sizes within *S. pyogenes* strain D471 cells undergoing enzymatic lysis owing to the action of PlyC, a holoenzyme composed of an octameric binding domain and a monomeric catalytic domain [25]. After the addition of the lysin, the lysing cells are chemically fixed to prevent changes in the cell wall hole sizes. The resulting images from ultrathin section transmission electron microscopy (TEM) were annotated with estimated diameters as shown in figure 3. Our estimate of the hole diameter is given by the width of the viewable aperture in the plane of the thin section. After screening dozens of thin sections containing thousands of cells, a total of 38 images were annotated in which membrane extrusions were visible in the plane of imaging (see the electronic supplementary material, file S1). The hole diameters range from 22 to 180 nm, with a mean of 68 and a 37 nm s.d. The smallest measured hole diameter was 22 nm, which is within the range of our prediction for the critical hole size. Thirty-seven of 38 (i.e. 97%) measured hole diameters exceeded the predicted upper cut-off for the critical hole size.

The results of these experiments can be compared with existing evidence for hole sizes across a range of bacteria strains and three different monomeric lysins compiled from the literature [37–39] which we present in table 1. Together with PlyC, these enzymes represent four distinct catalytic mechanisms that cleave different bonds in the peptidoglycan as follows: PlyC contains both *N*-acetylmuramoyl-l-alanine amidase and glycosyl hydrolase activities; PlyPH contains an *N*-acetylglucosaminidase activity; ClyS contains an endopeptidase activity; and Pal contains an *N*-acetylmuramoyl-l-alanine amidase activity. In every case, the observed hole sizes lie above the critical hole size predicted by theory and are in agreement with the range of hole sizes observed in our experiment. In summary, our theory predicts a range of critical hole diameters consistent with existing estimates of hole sizes in living cells and hole sizes in lysed cells.

**Table:** Table 1. Observed hole sizes of lysis events for distinct enzymes acting upon Gram-positive bacteria.

enzyme	bacteria	size (nm)	reference
Pal	*Streptococcus pneumonia*	36.8	Loeffler [37], figure 2*b*
		42.3	Loeffler [37], figure 2*c*
ClyS	*Staphylococcus aureus*	57.9	Daniel [38], figure 3*a*
		67.8	Daniel [38], figure 3*b*
		79.1	Daniel *et al*. [38]
		121.9	Daniel *et al*. [38]
		45.9	Daniel *et al*. [38], figure 3*c*
PlyPH	*Bacillus cereus*	46.1	Fischetti [39], figure 2a^a^

^a^Unpublished data associated with original publication.

## 5. Conclusion

We have developed and tested a biophysical theory of the response of Gram-positive bacteria to holes in their cell walls. We predict that cells should not lyse in the presence of small holes and will be susceptible to lysis in the presence of large holes. The theory predicts a range of hole sizes from 15 to 24 nm, below which holes are considered to be small, and above which holes are considered to be large. The balance between bending and pressure forces determines the critical hole range, which we validate by combining prior estimates of hole sizes in viable cells with novel experiments conducted to test the present theory. The combination of theory and experiments here provides insights into an important aspect of cell wall homeostasis and the biophysical mechanisms of enzymatic lysis. Previous efforts towards developing a quantitative understanding of this kind of lysis include detailed modelling of degradation of ‘vertically’ structured cell walls from without [40], the stochastic degradation of cell walls from within [41] and phenomenological models of the lysis from without in physiologically heterogeneous cultures [42]. The theoretical model developed here is the first to consider the effects of finite cell wall thickness on lysis. We predict that finite cell wall thickness does not impact the deterministic escape of the membrane with increasing hole size, and does very little to accelerate stochastic escape. For this reason, we suggest that the cell wall thickness may play a role in suppressing lysis by serving as a buffer against the formation of large holes. It is interesting to note that lysis events occur most often at the septal polls of the bacterial cell or at the junction between two cells growing in a chain. These points in the cell wall tend to have smaller relative thicknesses, and often lack cell wall associated teichoic acids and are not fully cross-linked [43,44]. All of these factors are likely to render these regions more susceptible to hole formation. Direct quantification of (i) hole formation owing to the action of enzymes and (ii) the membrane dynamics as a function of hole geometry remain as experimental challenges that would shed light on the fundamental mechanisms of lysis.

## Appendix A. Experimental material and methods

*Streptococcus pyogenes* strain D471 was grown overnight at 37°C in Todd–Hewitt media (Difco) supplemented with 1 per cent yeast extract. The next morning, cells were washed two times in sterile phosphate-buffered saline (pH 7.2), and exposed to 1 µg of PlyC, a streptococcal-specific cell wall hydrolase [45]. At 30 s, the reaction was stopped by cross-linking with 2 per cent glutaraldehyde in 0.1 M cacodylate buffer. Samples were then washed twice with cacodylate buffer, post-fixed with 2 per cent osmium tetroxide for 1 h, dehydrated with graded series of ethanol and embedded in Epon epoxy resin. Ultrathin sections (80 nm) were adsorbed in 300-mesh formvar/carbon-coated copper grids (Electron Microscopy Sciences), stained with 0.1 per cent lead citrate and 5 per cent uranyl acetate and examined by TEM, using a JEOL 1200 EX II electron microscope equipped with a 16 megapixel wide-angle bottom mount AMT digital camera (AMT16000M) for acquisition and processing of images. The annotated images showing estimated hole diameters are included as electronic supplementary material.

### Appendix B. Additional methods

Further explanation of the methodologies used here can be found in the electronic supplementary material, appendices. Appendix A derives in detail all the geometric quantities relevant to our discussion. Appendix B derives in detail the explicit forms for the energies and generalized forces. Electronic supplementary material, supplementary file S1 includes all 38 annotated images analysed in figure 3.
